# The value of circulating lymphocytic subpopulations in the diagnosis and repair of ischemic stroke patients with dizziness

**DOI:** 10.3389/fnagi.2022.1042123

**Published:** 2022-11-03

**Authors:** Yong Wang, Yichen Huang, Sicheng Li, Jixian Lin, Yang Liu, Yanqin Gao, Jing Zhao

**Affiliations:** ^1^Department of Neurology, Minhang Hospital, Fudan University, Shanghai, China; ^2^State Key Laboratory of Medical Neurobiology, MOE Frontier Center for Brain Science, and Institutes of Brain Science, Fudan University, Shanghai, China

**Keywords:** ischemic stroke, lymphocytes, neural repair, dizziness, biomarkers

## Abstract

**Background:**

To determine whether dizziness can contribute to stroke as a main cause still remains challenging. This study aims to explore clinical biomarkers in the identification of ischemic stroke patients from people with dizziness and the prediction of their long-term recovery.

**Methods:**

From January 2018 to June 2019, 21 ischemic stroke patients with a main complaint of dizziness, 84 non-stroke dizziness patients and 87 healthy volunteers were recruited in this study. Then, their peripheral blood samples were collected, and the percentages of circulating lymphocytes T cells, CD4^+^ T cells, CD8^+^ T cells, T^−/−^ cells (DNTs), CD4^+^ regulatory T cells (Tregs), CD8^+^ Tregs, B cells and regulatory B cells (Bregs) were examined to identify biomarkers with clinical value.

**Results:**

According to our data, a significant difference in the DNTs proportion was detected between non-stroke dizziness and ischemic stroke patients with dizziness (*p* = 0.0009). The Bregs proportion in ischemic stroke patients with dizziness was lower than that in non-stroke dizziness patients (*p* = 0.035). In addition, the percentage of Bregs and DNTs within lymphocytes in patients’ peripheral blood exhibited a significant negative correlation with stroke occurrence (Bregs, *p* = 0.039; DNTs, *p* = 0.046). Moreover, the Bregs and DNTs within lymphocytes were negatively related to participants’ age, while presented a weak relationship with clinical risks like smoking, hypertension, and diabetes. Then, area under the receiver operating characteristic curve (AUC) of Bregs and DNTs together was 0.768, the risk factors and Bregs or DNTs ranged from 0.795 and 0.792, respectively, and the AUC value of risk factors, Bregs and DNTs combination was further increased to 0.815. Furthermore, the Bregs percentage within lymphocytes at admission was also a potential predictor of repair at discharge and the following 3 months.

**Conclusion:**

Bregs and DNTs could be the clinical biomarkers together in the identification of ischemic stroke patients from people with dizziness.

## Introduction

As a major cause of mortality and morbidity worldwide, stroke has been prioritized by World Health Organization in their efforts to reduce the burden of non-communicable diseases (Feigin et al., 2015, 2017). Stroke chameleons is a situation in which it is manifested by atypical stroke symptoms as opposed to hemiparesis, facial droop, or dysarthria (Arch et al., 2016; Venkat et al., 2018; Moulin and Leys, 2019). As one of the most common symptoms among stroke chameleons, dizziness is responsible for an estimated 4.4 million visits of emergency department in American annually, while about only 3%–5% of such visits are attributed to stroke (Saber Tehrani et al., 2013; Newman-Toker and Edlow, 2015). This situation is usually associated with a high cost and medical resource expenditure, because of neuroimaging examination in roughly half the patients (Newman-Toker et al., 2013b; Newman-Toker, 2016), high misdiagnosis rate of posterior circulation stroke with dizziness and the great impact on patients’ prognosis (Liberman and Prabhakaran, 2017). Currently, intravenous alteplase and mechanical thrombectomy are two recommended effective treatments for ischemic stroke patients (Yaghi et al., 2017; Munich et al., 2019). However, these two treatments are highly time-sensitive (Saver et al., 2016). Failure of correct stroke diagnosis in time may place barriers in the initiation of secondary stroke prevention, thus missing the opportunity to avoid a poor prognosis (Richoz et al., 2015). Therefore, an evidence-based and cost-effective approach to distinguish stroke categories is needed.

The immune response plays a key role in the pathophysiology of ischemic stroke (Macrez et al., 2011; Li et al., 2018). Previous studies have proved that the pathophysiological process of ischemic stroke is not only involved inflammatory in local brain, but also related to that in the peripheral inflammatory system (Buck et al., 2008; McColl et al., 2009). Additionally, markers of immune activation were observed in the patients’ peripheral blood after stroke, and activated lymphocytes were found in peripheral blood before presenting in the brain (Urra et al., 2009; Yan et al., 2009). Furthermore, the local inflammatory changes in the central nervous system of stroke patients can be reflected by the dynamic change of peripheral blood lymphocyte subpopulations (Guoping et al., 2015). At present, peripheral blood lymphocyte subpopulations have been reported to be predictors of short-term and long-term stroke outcomes (Li et al., 2021). However, there is no study on the value of lymphocyte subpopulations in the identification of cerebral infarction in patients with dizziness as the main complaint.

Herein, the aim of the present study is to analyze the characteristics of non-stroke dizziness patients and stroke patients with dizziness, and investigate the difference of the peripheral blood lymphocyte subpopulations in the two groups for reliable biomarkers in the identification of ischemic stroke patients from all dizziness patients.

## Materials and methods

### Ethics and participants

The clinical study was approved by the Ethical Review Board of Central Hospital of Minhang District (Shanghai, China). From January 2018 to June 2019, 21 stroke patients with a chief complaint of dizziness, and 84 patients with non-stroke dizziness were included according to following criteria: (1) more than 18 years old; (2) stroke patients with a final diagnosis of “cerebral infarction” confirmed by head CT or MRI; (3) non-stroke dizziness patients with a final diagnosis of “dizziness and vertigo”; (4) first onset; (5) onset time no more than 5 days; (6) informed consent was obtained from the patients or their legal representatives. Patients with brain hemorrhage, neoplasms, or acute infections after stroke were excluded. Clinical information of stroke patients was collected at admission, including age, gender, risk factors (hypertension, diabetes, disorder of lipid metabolism, atrial fibrillation, smoking, alcohol consumption, and cerebral infarction history). Control subjects were 87 age- and gender-matched healthy volunteers without a history of stroke or other vascular diseases.

### Isolation of peripheral blood mononuclear cells

After the informed consent was obtained from participants or their legal representatives, peripheral blood samples were collected through forearm veins from the participants, and Ethylene Diamine Tetraacetic Acid (EDTA) vacutainer blood collection tube was used to keep those samples. For non-stroke dizziness and stroke patients, blood samples were collected within 24 h from admission. For healthy volunteers, blood samples were collected when they attended the physical examination. PBMCs were isolated by density gradient centrifugation within 3 h after receipt of peripheral blood samples. Technically, the blood samples were firstly diluted in a 1:1 ratio with PBS buffer, and then layered over Ficoll-Paque (GE Healthcare Bio Sciences) in a 15 ml centrifuge tube. After 30 min of centrifugation at 400*g* and 20°C, PBMCs were finally collected from the plasma-Ficoll interphase.

### Flow cytometry analysis

Trypan blue exclusion method was used to quantify PBMCs, then 10^6^–10^7^ cells were labeled with fluorochrome-conjugated antibodies (BD Biosciences), including FITC Mouse Anti-Human CD3, APC-H7 Mouse Anti-Human CD4, APC Mouse Anti-Human CD25, Percp-cy5.5 Mouse Anti-Human CD19, PE Mouse Anti-Human CD24, PE Mouse Anti-Human CD127, APC Mouse Anti-Human CD27, Percp-cy5.5 Mouse Anti-Human CD8, PE Mouse Anti-Human CD45RA, APC Mouse Anti-Human CD62L, and PE-cy7 Mouse Anti-Human CD183. All samples were run on LSRFortessa (BD Biosciences) using FACSDiva software (version 8.0), and FlowJo (version 9.9.6) was used to analyze the data.

### Statistical analysis

Patient characteristics and experimental data were presented as means ± standard deviations (SDs) or numbers (%). Correlations between cell proportion and multiple characteristics were analyzed using Mann–Whitney *U* test and Spearman’s correlation analysis as appropriate. The statistical significance of differences between experimental groups was assessed with the Brown-Forsythe and Welch ANOVA tests. All above statistical analyses were performed with GraphPad prism software (version 9.0). A forward stepwise binary logistic regression was performed with SPSS 25.0 software package (SPSS Inc., Chicago, Illinois). Variables showing significant association with stroke occurrence in univariate analysis (*p* < 0.1) and important confounders were included in the multivariate logistic regression analysis. Area under the receiver operating characteristic curve (AUC) was calculated and visualized with R-3.5.2 software^1^ using R package *pROC* (receiver operating characteristic curve). If not specified above, a two-tailed *p* < 0.05 was considered statistically significant.

## Results

### Clinical characteristics of participants

A total of 105 patients and 87 healthy controls were enrolled in our study, whose demographic characteristics were described in Table 1. No statistically significant difference was observed in either age or sex between the controls and patients. Among the 105 dizziness patients, 21 patients were diagnosed with ischemic stroke and 84 with dizziness and vertigo. The clinical characteristics of all patients were demonstrated in Table 2. There was no significant group difference between the 21 stroke patients (3 ± 1.2 days) and 84 non-stroke dizziness patients (2 ± 1.2 days) from the onset to admission time. All the neurological scores of stroke patients were recorded at admission by National Institutes of Health Stroke Scale (NIHSS), modified Rankin Scale (mRS), Glasgow Coma Scale (GCS). Additionally, all patients’ risk factors including hypertension, diabetes, smoking and alcohol consumption were also recorded. The data showed that no statistically difference was observed between the stroke and non-stroke dizziness patients in the history of various risk factors, including hypertension (*p* = 0.23), diabetes (*p* = 0.30), smoking (*p* = 0.08), and alcohol consumption (*p* = 0.17). Furthermore, no significant group difference existed between the stroke patients and non-stroke dizziness patients in the laboratory tests, namely white blood cell count (*p* = 0.42), neutrophil proportion (*p* = 0.42), lymphocyte proportion (*p* = 0.43), monocyte proportion (*p* = 0.05), eosinophil granulocyte proportion (*p* = 0.84) and basophil granulocyte proportion (*p* = 0.72). The specific final diagnosis and detailed radiological characteristics of stroke patients were described in Supplementary Table 1.

**Table 1:** Age and gender differences between patients and controls.

	Stroke (*n* = 21)	Non-stroke dizziness (*n* = 84)	Control (*n* = 87)	Value of *P*
Age, years, M ± SD	66 ± 15	69 ± 12	64 ± 14	0.103
Sex, Male, *N* (%)	10 (47.62%)	34 (40.48%)	50 (57.47%)	0.084

One-way ANOVA test and Pearson Chi-Square test were employed to examine the differences in age and sex between controls and patients as appropriate. SD, standard deviation.

**Table 2:** Clinical characteristics of patients.

	Stroke (*n* = 21)	Non-stroke dizziness (*n* = 84)	Value of *p*
Onset to admission time, days, mean ± SD	3 ± 1.2	2 ± 1.2	0.401
NIHSS, mean ± SD	2.10 ± 2.70	–	–
mRS, mean ± SD	1.33 ± 0.856	–	–
GCS, mean ± SD	14.95 ± 0.218	–	–
SBP, mmHg mean ± SD	125.22 ± 19.188	–	–
DBP, mmHg mean ± SD	80.94 ± 9.471	–	–
Specific final diagnosis		–	–
Cerebral infarction	21 (100%)	–	–
Dizziness and vertigo	–	84 (100%)	–
Risk factors			
Hypertension, *n* (%)	11 (52.38%)	32 (38.95%)	0.234
Diabetes, *n* (%)	4 (19.05%)	7 (8.33%)	0.300
Disorder of lipid metabolism, *n* (%)	3 (14.29%)	–	–
Atrial fibrillation, *n* (%)	3 (14.29%)	–	–
Smoking, *n* (%)	4 (19.05%)	4 (4.76%)	0.081
Alcohol consumption, *n* (%)	3 (14.29%)	3 (3.57%)	0.172
Cerebral infarction history, *n* (%)	4 (19.05%)	–	–
Laboratory studies			
WBC count, mean ± SD * 109	7.249 ± 2.116	6.799 ± 2.138	0.416
Neutrophil %, mean ± SD	59.681 ± 6.399	61.70 ± 9.534	0.422
Lymphocyte %, mean ± SD	30.46 ± 9.497	28.66 ± 8.628	0.432
Monocyte %, mean ± SD	7.589 ± 2.026	6.693 ± 1.664	0.0502
Eosinophil granulocyte %, mean ± SD	1.733 ± 1.199	1.677 ± 1.005	0.840
Basophil granulocyte %, mean ± SD	0.211 ± 0.129	0.197 ± 0.144	0.715

Mann–Whitney *U* test, Chi-Square test and Independent-sample *T* test were employed to examine the differences in onset to admission time, risk factors and laboratory studies between controls and patients as appropriate. NIHSS, National Institutes of Health Stroke Scale; mRS, modified Rankin Scale; GCS, Glasgow Coma Scale; WBC, white blood cell.

### Changes of lymphocytic subpopulations in different groups

**Figure 1:**
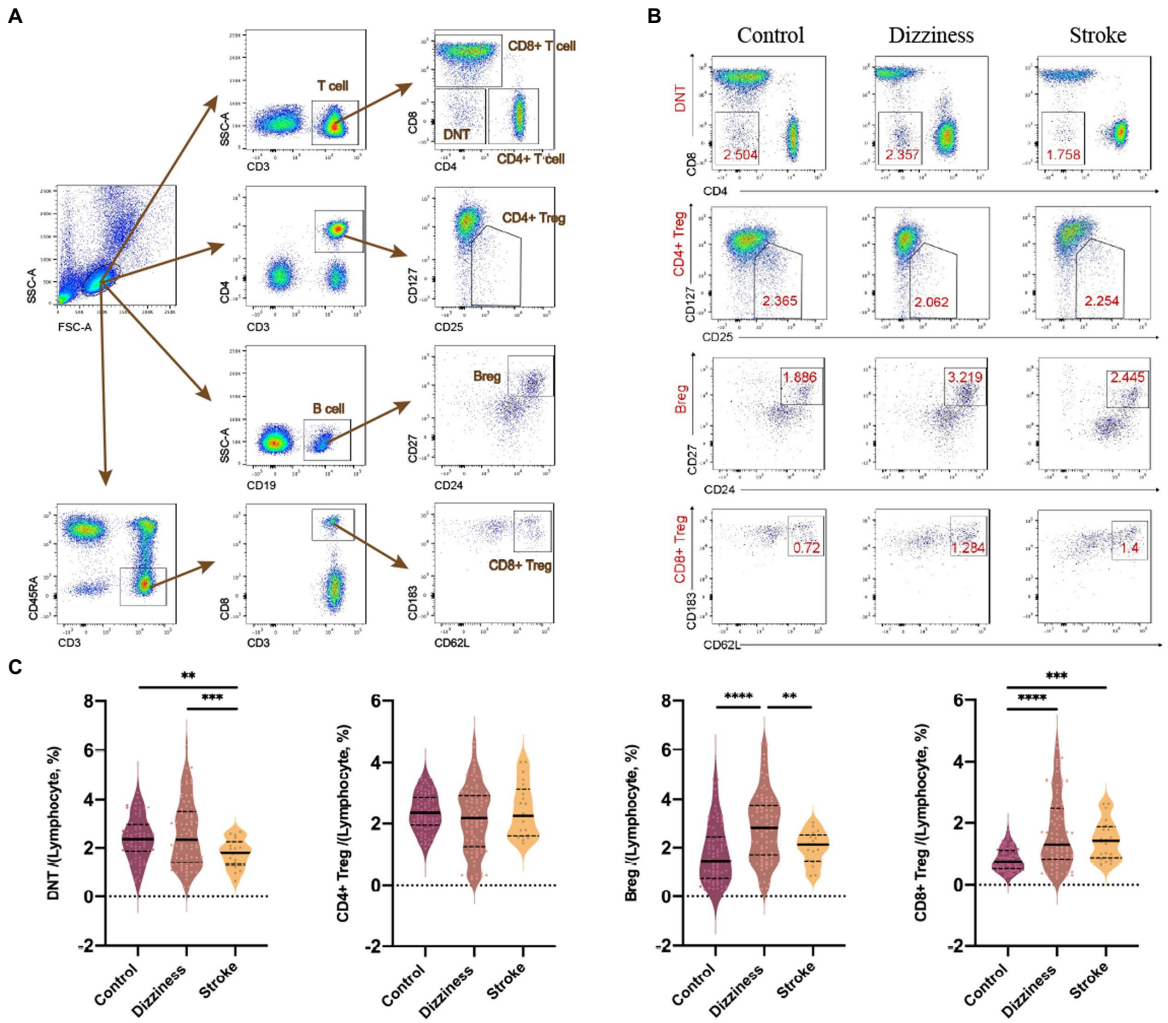
Changes of lymphocytic subpopulations in the control, dizziness and stroke groups. **(A)** Representative flow cytometry analyses on the total lymphocytes, B cells, T cells, CD4+ T cells, CD8+ T cells, Bregs, CD4+ Tregs, CD8+ Tregs, and DNTs. **(B)** Representative flow cytometry plot of DNTs, CD4+ Tregs, Bregs, and CD8+ Tregs in peripheral blood of control, dizziness, and stroke groups. **(C)** Violin plots of DNTs, CD4+ Tregs, Bregs, and CD8+ Tregs within lymphocytes of control (*n* = 87), dizziness (*n* = 84), and stroke (*n* = 21). ^**^p < 0.01, ^***^p < 0.001, ^****^p < 0.0001.

With the use of flow cytometry, different lymphocytic subpopulations among all controls, non-stroke dizziness and stroke patients were analyzed, including CD19^+^ cells (B cells), CD3^+^ cells (T cells), CD3^+^CD4^+^ cells (CD4^+^ T cells), CD3^+^CD8^+^ cells (CD8^+^ T cells), CD3^+^CD4^−^CD8^−^ cells (Double Negative T cells, DNTs), and immunoregulatory lymphocytes, namely CD19^+^CD24^+^CD27^+^ cells (Bregs), CD3^+^CD4^+^CD25^+^CD127^−^ cells (CD4^+^ Tregs) and CD3^+^CD45RA^−^CD8^+^CD183^+^CD62L^+^ cells (CD8^+^ Tregs, Figure 1A). It was found that no significant group difference was observed between the non-stroke dizziness and stroke patients in the aspects of B cells (12.1 ± 5.54 vs.12.4 ± 5.38, *p* = 0.995), T cells (64.4 ± 9.25 vs. 65.3 ± 12.72, *p* = 0.98), CD4^+^ T cells (37.3 ± 9.77 vs. 39.8 ± 13.55, *p* = 0.80) and CD8^+^ T cells (20.6 ± 8.02 vs. 19.3 ± 7.44, *p* = 0.86, Supplementary Figure 1). The cell proportion analysis of DNTs indicated a significant difference between non-stroke dizziness and stroke patients (2.6 ± 1.32 vs.1.8 ± 0.59, *p* = 0.0009), while there was no statistically difference between non-stroke dizziness patients and health controls (2.6 ± 1.32 vs. 2.4 ± 0.88, *p* = 0.59; Figures 1B,C). Besides, the immunoregulatory lymphocytes analysis indicated that the cell proportion of Bregs in stroke patients was lower than that in non-stroke dizziness patients (2.9 ± 1.41 vs. 2.0 ± 0.66, *p* = 0.035). However, there was no group difference found in CD4^+^ Tregs (2.2 ± 1.18 vs. 2.4 ± 0.87, *p* = 0.53) and CD8^+^ Tregs (1.7 ± 1.14 vs. 1.5 ± 0.65, *p* = 0.56; Figures 1B,C).

### Relationships between the lymphocytic subpopulations and stroke

To further explore the potential value of regulatory lymphocytes in distinguishing stroke patients from dizziness patients, 5 independent variables (Bregs, DNTs, age, gender and smoking) were included in the forward stepwise binary logistic regression analyses. As shown in the Table 3, among the 5 independent variables, Bregs and DNTs had statistical significance with the stroke occurrence, while age, gender and smoking did not. The percentage of Bregs within lymphocytes in patients’ peripheral blood samples exhibited a significant negative relationship with stroke occurrence (*p* = 0.04). Similarly, the percentage of DNTs within lymphocytes also showed a significant negative relationship with stroke occurrence (*p* = 0.05). These results suggested that the lower level of Bregs or DNTs in dizziness patients at admission, the higher likelihood of stroke occurrence.

**Table 3:** Logistic regression.

Variables	OR (95% CI)	Value of *p*
Bregs	0.586 (0.352–0.973)	0.039
DNTs	0.550 (0.306–0.989)	0.046
Age	–	0.253
Gender	–	0.958
Smoking	–	0.458

Data were analyzed using forward stepwise binary logistic regression.

### Relationships between lymphocytic subpopulations and clinical risks

The occurrence and progression of stroke are associated with multiple factors, including age, gender, and basic diseases. Blood leukocytes are also known to be influenced by a series of diseases or lifestyle habits. Thus, the relationships between regulatory lymphocytes and clinical risks were further assessed in order to clarify the dependability of Bregs and/or DNTs as biomarkers in predicting stroke occurrence. As shown in the Table 4, the percentage of Bregs and DNTs within lymphocytes in the healthy controls were negatively related to age, not gender difference. Similarly, the percentages of Bregs in the non-stroke dizziness patients and DNTs in the stroke patients were negatively related to age, not gender difference. Additionally, Bregs and DNTs presented a weak relationship with clinical risks like smoking, hypertension, and diabetes. It was found that only the percentage of Bregs in the non-stroke dizziness patients showed a significant difference in alcohol consumption. Non-stroke dizziness patients with alcohol consumption habit had significant lower Bregs than those without alcohol consumption habit. These results indicate that Bregs and DNTs are independent with most clinical risks, which proves their dependability as clinical biomarkers in stroke.

**Table 4:** Relationships between regulatory lymphocytes and clinical risks.

	Mann–Whitney *U* test			Linear regression	
	Median		Value of *p*	Spearman *r*	Value of *p*
Health	Sex			Age	
	Male	Female			
Bregs	1.3921	1.9790	0.058	−0.724	<0.001
DNTs	2.6839	2.3465	0.101	−0.264	0.013
Non-stroke dizziness					
Bregs	2.5161	3.2492	0.073	−0.262	0.016
DNTs	2.8351	2.4265	0.993	−0.049	0.657
Stroke					
Bregs	2.2158	2.3867	0.833	−0.081	0.728
DNTs	1.9860	2.0162	0.526	−0.453	0.039
	**Alcohol consumption**				
**Non-stroke dizziness**	**No**	**Yes**	**Value of *p***		
Bregs	3.0503	1.3500	0.024		
DNTs	2.5375	3.4825	0.638		
Stroke					
Bregs	2.2158	2.6938	0.421		
DNTs	2.0041	1.3988	0.421		
	**Smoking**				
**Non-stroke dizziness**	**No**	**Yes**	**Value of *p***		
Bregs	3.0513	1.9293	0.106		
DNTs	2.6017	1.2586	0.172		
Stroke					
Bregs	2.2743	2.2784	0.858		
DNTs	2.0162	1.3140	0.179		
	**Hypertension**				
**Non-stroke dizziness**	**No**	**Yes**	**Value of *p***		
Bregs	3.2216	2.6917	0.490		
DNTs	2.5794	2.3521	0.242		
Stroke					
Bregs	2.2158	2.3867	0.778		
DNTs	2.3132	1.9800	0.481		
	**Diabetes**				
**Non-stroke dizziness**	**No**	**Yes**	**Value of *p***		
Bregs	2.8853	3.6879	0.577		
DNTs	2.5375	3.4825	0.789		
Stroke					
Bregs	2.2743	1.9003	0.420		
DNTs	2.0923	1.4335	0.089		

Data were analyzed using Mann–Whitney *U* test and lineal regression. In Mann–Whitney *U* test, data were presented as median.

### The dependability of Bregs and DNTs in stroke diagnosis

**Figure 2:**
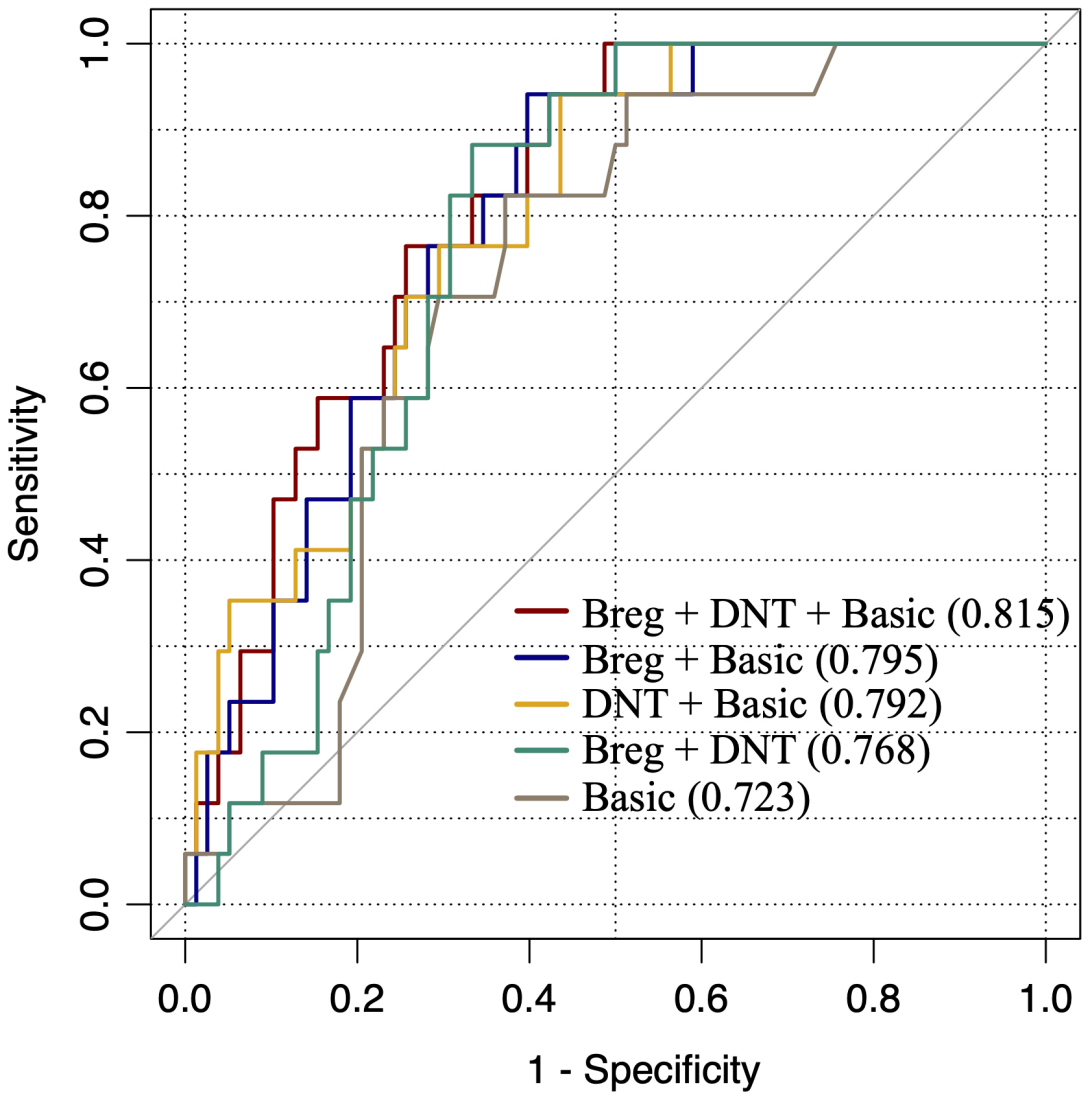
ROC curves to assess dependability of lymphocytic subpopulations in distinguishing stroke patients from dizziness patients. “Basic”: factors including gender, age, past history of hypertension, diabetes and habits of alcohol consumption and smoking; “Breg+DNT + Basic”: “Basic” adding DNTs/lymphocytes and Bregs/lymphocytes; “Breg+Basic”: “Basic” adding Bregs/lymphocytes; “DNT + Basic,” “Basic” adding DNTs/lymphocytes; “Breg+DNT”: Bregs/lymphocytes adding DNTs/lymphocytes.

In order to evaluate the dependability of Bregs and DNTs in distinguishing stroke patients from non-stroke dizziness patients, the percentages of these two cells along with the clinical features were used to calculate ROC curves and AUC. The AUC of the Bregs and DNTs combination was 0.77, which was larger than that of clinical risk factors that related to stroke including age, gender, past history of hypertension, diabetes, habits of alcohol consumption and smoking (AUC = 0.72). Additionally, the AUC values of risk factors combined with Bregs or DNTs were 0.795 and 0.792, respectively. Moreover, the combination of Bregs, DNTs and clinical risk factors further elevated the AUC to 0.82 (Figure 2). These suggested that Bregs and DNTs could be used to facilitate the diagnosis of ischemic stroke in dizziness patients.

### Relationships between lymphocytic subpopulations and neural repair

The values of Bregs and DNTs in predicting neural repair of stroke patients were further evaluated by Pearson correlation analysis (Table 5). The results suggested that stroke patients with higher percentage of Bregs within lymphocytes at admission had better neural repair at discharge [NIHSS: *R* = −0.45, *p* = 0.04; mRS: *R* = −0.5, *p* = 0.04] and the 3rd month [NIHSS: *R* = −0.62, *p* = 0.003; mRS: *R* = −0.36, *p* = 0.105], while DNTs were not related the repair process.

**Table 5:** Relationships between regulatory lymphocytes and neural repair.

Admission (/lymphocytes, %)	Discharge				3 months			
	NIHSS		mRS		NIHSS		mRS	
	*R*	Value of *p*	*R*	Value of *p*	*R*	Value of *p*	*R*	Value of *p*
Bregs	−0.447	0.042	−0.45	0.041	−0.618	0.003	−0.364	0.105
DNTs	0.196	0.396	0.184	0.424	0.141	0.542	0.166	0.472

Data were analyzed using Pearson correlation. *R*, Pearson correlation coefficient; NIHSS, National Institutes of Health Stroke Scale; mRS, modified Rankin Scale.

## Discussion

According to previous studies, dizziness is a common reason for the emergency department (ED) visit, and stroke patients only account for about 3%–5% of all ED patients with dizziness (Newman-Toker and Edlow, 2015). Accurate screening of potential stroke patients from all dizziness patients is of paramount importance to ensure timely and appropriate treatment (Newman-Toker et al., 2013a,c). However, the identification of this small subset of stroke patients from dizziness patients represents a great challenge, and cost a large amount of medical resource (Lapchak and Zhang, 2017). The HINTS exam (Head Impulse, Nystagmus, Test of Skew) is a useful clinical tool with a series of ocular motor tests to discriminate the central and peripheral causes of dizziness (Newman-Toker and Edlow, 2015; Zwergal and Dieterich, 2020). Nevertheless, HINTS test is not appropriate for patients with acute transient vestibular syndrome, as the typical vestibular symptoms had already disappeared when they arrived at the hospital (Choi et al., 2017). Additionally, the HINTS test is of limited utility in ED without a neuro-ophthalmologist, as most ED physicians may be out of the ability to identify appropriate candidates and interpret the results of the exam (Choi et al., 2018; Dmitriew et al., 2021). Besides, the diffusion-weighted imaging sequence in MRI plays an important role in brain examination, and it has been proven to be sensitive in detecting cerebral infarction (Chalela et al., 2007). However, it is not cost-effective to take MRI scanning in all dizziness patients in order to screen out the suspected cerebral infarction patients (Wardlaw et al., 2004). Furthermore, it may not always be available for all patients to receive an MRI scan (Wardlaw et al., 2004). Therefore, it is necessary to find a reliable biomarker to assist with existing tests like HINTS, or to screen out most probable ischemic stroke patients who require further head MRI examination from all dizziness patients.

Routine blood test is an easy and convenient test in clinical practice. In the present study, 84 non-stroke dizziness patients and 21 stroke patients with dizziness were recruited, and the clinical data including white blood cells count, percentage of neutrophils, lymphocytes, monocytes, eosinophils and basophils were collected for further analysis. Although a significant difference of these inflammatory cells was found between non-stroke dizziness patients and controls, and stroke patients and controls, no significance difference existed between non-stroke dizziness patients and stroke patients. These results revealed that these inflammatory cells could reflect the unhealthy state of patients, but were unable to distinguish non-stroke dizziness and stroke dizziness. Then, we further collected the peripheral blood samples of patients within 24 h at admission after onset, and used flow cytometry to detect the change of different lymphocytic subpopulations. Notably, the changes in lymphocytic subpopulations were significant. Not only the differences of B cells, Bregs, CD8^+^ Tregs and DNTs between patients and controls, but also the differences of Bregs and DNTs between non-stroke dizziness and stroke groups were statistically significant.

Bregs are a subset of B cells that secrete interleukin-10 (IL-10; Seifert et al., 2018). Recent study has proven that Bregs can limit central nervous system inflammation in experimental stroke model, which benefits stroke patients (Bodhankar et al., 2013). In the present study, the level of Bregs was decreased in stroke patients, which was consistent with the previous study (Li et al., 2021). The Bregs level of non-stroke dizziness patients was higher than that of stroke patients, which indicated that Bregs could be a potential biomarker for distinguishing dizziness and stroke. DNTs are a small subset of T cells with diverse roles in peripheral immune-related diseases, as they can regulate immunological and inflammatory homeostasis (Ford et al., 2007; D'Acquisto and Crompton, 2011). The sources of DNTs in the diseased brain of ischemic stroke patients are still unknown, and few studies focus on the roles of DNTs in the brain. Our study found that the percentage of DNTs within lymphocytes was significant decreased in stroke patients, consistent with the previous study (Li et al., 2021). These results indicated that DNTs could be a suitable biomarker for clinical physicians to differentiate non-stroke dizziness and stroke, because it was normal in non-stroke dizziness patients but decreased in stroke patients with dizziness. The change of the DNTs level is differ from that in the previous study (Meng et al., 2019), but it could be clearly explained. Firstly, the previous study with all 47 stroke patients ignores their chief complaint, while only stroke patients with dizziness as the main complaint are enrolled in our study. Therefore, the clinical characteristics were different in these two populations. Secondly, the elevated level of DNTs is observed only in 68.09% (32/47) of stroke patients, instead of all 47 patients in the previous study, so we cannot exclude the possibility that some stroke patients show decreased level of DNTs.

Further, we explored the relationship of regulatory lymphocytes, stroke occurrence and clinical risks. The results indicated that both Bregs and DNTs exhibited a significant negative relationship with stroke occurrence. It means that the lower the level of Bregs or DNTs, the higher the risk of stroke in those patients with a chief complaint of dizziness. Additionally, Bregs or DNTs had a weak correlation with smoking, hypertension and diabetes, while they were closely related to age and alcohol consumption. As alcohol consumption status was unrecorded in controls and the percentage of alcohol consumption person was very low in both non-stroke dizziness and stoke patients, their relationship was not further explored. As for age, Bregs and DNTs showed a negative relationship with it, which indicates that young dizziness patients with low level of Bregs and/or DNTs are high-risk group. In addition, we assessed the abilities of Bregs and DNTs in predicting neural repair of stroke patients. The results indicated that the percentage of Bregs within lymphocytes at admission was a potential predictor of neural repair at discharge and the following 3 months. Further studies are needed to investigate the underlying mechanisms of Bregs in affecting the long-term prognosis of ischemic stroke patients.

Next, ROC curves reveled that both Bregs and DNTs combined with the clinical risk factors can be used to distinguish non-stroke dizziness and stroke patients with dizziness to some extent. Previous studies indicated that clinical risk factors related to stroke included age, gender, past history of hypertension, diabetes and habits of alcohol consumption and smoking (Aigner et al., 2017; Qi et al., 2020). In present study, the AUC of these risk factors was 0.723, showing a moderate quality for discriminating stroke from non-stroke dizziness, consistent to the views of previous study (Aigner et al., 2017). The AUC of Bregs and DNTs combination was 0.768 with a moderate specificity and a good sensitivity, which was larger than that of risk factors. It indicates that the dependability of Bregs and DNTs in distinguishing stroke from non-stroke dizziness is better than risk factor to a certain extent, and the high sensitivity of this model is more conducive to screening potential stroke patients. We further evaluated the AUC values when Bregs or DNTs combined with risk factors. The results showed that both Bregs and DNTs could improve the AUC of basic model significantly. However, the combination of Bregs and DNTs can improve the AUC of basic model to 0.815, which makes it a high-quality model and indicates the great utility value of Bregs and DNTs. Therefore, the present study suggests that Bregs and DNTs possess a good ability to assess the occurrence of ischemic stroke among patients with a chief complaint of dizziness. The application of these peripheral blood markers would be beneficial for screening potential posterior circulation infarction patients effectively, which could not be limited by under-developed equipments of grassroots hospitals. At present, it is still difficult to apply these biomarkers in clinic, but they have great application value as the technology improves. Additionally, further studies are needed to investigate the roles, cooperation and antagonism of these regulatory lymphocytes in ischemic stroke patients to clarify the underlying mechanisms and facilitate the novel targets search for treatment.

This study has some limitations. First, only lymphocyte levels in the peripheral blood were analyzed in the present study, not including lymphocyte levels in the brain. The evaluation of peripheral markers can mask events coming from the central nervous system due to the contamination from systemic sources. Second, the stroke risk factors of healthy persons in control group are not recorded as they are volunteers without intact medical records. Third, the sample size of our study is small. Prospective studies with larger samples are needed to further validate our findings.

## Conclusion

Bregs and DNTs can be the clinical biomarkers for the diagnosis of ischemic stroke among dizziness patients, and Bregs could also be a potential predictor of neural repair. These findings will lay scientific foundation for Bregs and DNTs application in clinic, which facilitates the diagnosis and subsequent recovery of ischemic stroke patients.

## Data availability statement

The raw data supporting the conclusions of this article will be made available by the authors, without undue reservation.

## Ethics statement

The studies involving human participants were reviewed and approved by The Ethical Review Board of Central Hospital of Minhang District (Shanghai, China). The patients/participants provided their written informed consent to participate in this study.

## Author contributions

YW, JZ, and YG conceived and designed the experiments. YH and SL performed the experiments. YW and JL analyzed the data. YW and YL prepared the figures. YW wrote the paper. YW and YH revised the manuscript. All authors contributed to the article and approved the submitted version.

## Funding

This work was supported by the National Nature Science Foundation of China (grant nos. 81973157, 82173646, and 81870971), the Chinese State Ministry of Science and Technology grants (2021ZD0201704), and Supported by Shanghai Municipal Science and Technology Major Projects (22ZR1413700 and 2018SHZDZX01), and Shanghai Center for Brain Science and Brain-Inspired Technology.

## Conflict of interest

The authors declare that the research was conducted in the absence of any commercial or financial relationships that could be construed as a potential conflict of interest.

## Publisher’s note

All claims expressed in this article are solely those of the authors and do not necessarily represent those of their affiliated organizations, or those of the publisher, the editors and the reviewers. Any product that may be evaluated in this article, or claim that may be made by its manufacturer, is not guaranteed or endorsed by the publisher.

## Footnotes

^1^ https://www.r-project.org/
