# Supplementary material for: The value of circulating lymphocytic subpopulations in the diagnosis and repair of ischemic stroke patients with dizziness
Source: Front Aging Neurosci. 2022 Nov 3;14:1042123. doi: 10.3389/fnagi.2022.1042123 (PMC9670111; doi:10.3389/fnagi.2022.1042123)
Supplement: Supplementary file 1 [file Data_Sheet_1.PDF]

Supplementary Material

1.1 Supplementary Figures

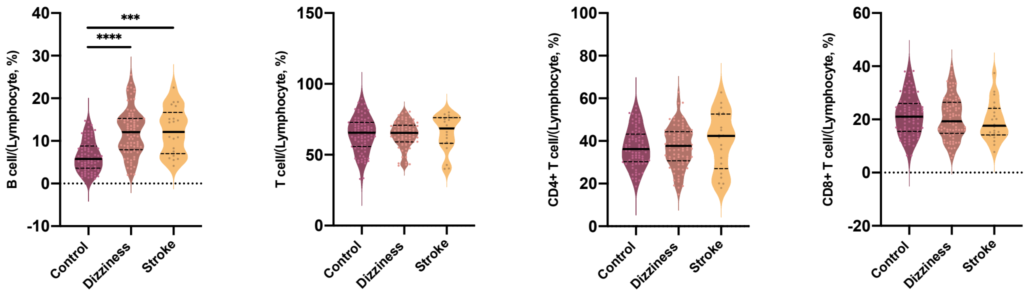

Supplementary Figure 1. Violin plots with median, 25 and 75% quartiles of the percentage of B cells, T cells, CD4+ T cells, and CD8+ T cells within lymphocytes of Control (n = 87), non-stroke dizziness (n = 84), and stroke (n = 21). ****: $p < 0.0001$.

1.2 Supplementary tables

Table 1. Clinical and radiological characteristics of stroke patients

Patients	Diagnosis	TOAST classification	Infarct location	Infarct volume (cm^3)
1	cerebral infarction	SUE	Bilateral cerebellar hemisphere subacute cerebral infarction	14.64
2	cerebral infarction	LAA	Subacute cerebral infarction in the right cerebellar hemisphere and left basal ganglia	6.797
3	cerebral infarction	LAA	Subacute cerebral infarction in the right basal ganglia	1.08
4	cerebral infarction	SUE	Subacute infarcts in the left basal ganglia	0.4965
5	cerebral infarction	LAA	Subacute cerebral infarction of the right temporal lobe	3.39
6	cerebral infarction	SAA	Bilateral basal ganglia, lateral ventricle and marginal parietal white matter multiple lacunar infarction (from CT)	No MRI scan

7	cerebral infarction	CE	Right occipital lobe cerebral infarction with hemorrhage	0.056
8	cerebral infarction	LAA	Bilateral ventricle and semi-oval area scattered in ischemic foci	0.062
9	Cerebral infarction	LAA	Brain stem subacute cerebral infarction	0.775
10	cerebral infarction	LAA	The right frontal temporal lobe is scattered in a subacute infarction	0.063
11	cerebral infarction	LAA	Subacute cerebral infarction on the left occipital lobe, which tends to be chronic	0.056
12	cerebral infarction	LAA	Bilateral ventricle and semi-oval area scattered in ischemic foci	0.0125
13	cerebral infarction	SUE	The right frontal parietal cortex and cortex are scattered in a small subacute infarct	1.234
14	cerebral infarction	SAA	Bilateral basal ganglia, lateral ventricle and marginal parietal white matter multiple lacunar infarction (from CT)	No MRI scan
15	cerebral infarction	SUE	Right cerebellar hemisphere subacute lacunar infarction	0.1345
16	cerebral infarction	LAA	Cervical stem, bilateral basal ganglia, lateral ventricle and semi-oval area with multiple lacunar infarction and ischemic foci	0.0078
17	cerebral infarction	LAA	Right cerebellar hemisphere subacute cerebral infarction, medullary right margin cerebral infarction (subacute-chronic)	1.0346
18	cerebral infarction	SUE	Right occipital softening lesions with peripheral glial hyperplasia	0.024
19	cerebral infarction	LAA	Bilateral occipital subacute cerebral infarction	9.63
20	cerebral infarction	SAA	Bilateral basal ganglia, lateral ventricle and frontal parietal white matter multiple lacunar infarction and ischemic foci	0.054
21	cerebral infarction	LAA	Brain stem, bilateral basal ganglia, lateral ventricle and semi-oval area with multiple ischemic lesions	0.316

Note: SUE, stroke of undetermined etiologies; LAA, large artery atherosclerosis; SAA, small artery occlusion or lacunar stroke; CE, cardio embolic disease.
